# Effect of Standard Tuberculosis Treatment on Plasma Cytokine Levels in Patients with Active Pulmonary Tuberculosis

**DOI:** 10.1371/journal.pone.0036886

**Published:** 2012-05-14

**Authors:** Catherine Riou, Blas Perez Peixoto, Lindi Roberts, Katharina Ronacher, Gerhard Walzl, Claudia Manca, Roxana Rustomjee, Thuli Mthiyane, Dorothy Fallows, Clive M. Gray, Gilla Kaplan

**Affiliations:** 1 Division of Immunology, Institute of Infectious Diseases and Molecular Medicine (IIDMM), Clinical Laboratory Sciences, University of Cape Town, Cape Town, Western Cape, South Africa; 2 Laboratory of Mycobacterial Immunity and Pathogenesis and the TB Research Unit (TBRU), Public Health Research Institute at the University of Medicine and Dentistry of New Jersey (UMDNJ), Newark, New Jersey, United States of America; 3 Division of Medical Virology, Institute of Infectious Diseases and Molecular Medicine (IIDMM), University of Cape Town, Cape Town, Western Cape, South Africa; 4 Department of Biomedical Sciences, Department of Science and Technology/National Research Foundation (DST/NRF) Center of Excellence for TB Research, Stellenbosch University, Cape Town, Western Cape, South Africa; 5 Tuberculosis Research Unit - Clinical and Biomedical, Medical Research Council, Durban, KwaZulu-Natal, South Africa; National Institute for Infectious Diseases (L. Spallanzani), Italy

## Abstract

**Background:**

Sputum *Mycobacterium tuberculosis* (Mtb) culture is commonly used to assess response to antibiotic treatment in individuals with pulmonary tuberculosis (TB). Such techniques are constrained by the slow growth rate of Mtb, and more sensitive methods to monitor Mtb clearance are needed. The goal of this study was to evaluate changes in plasma cytokines in patients undergoing treatment for TB as a means of identifying candidate host markers associated with microbiologic response to therapy.

**Methods:**

Twenty-four plasma cytokines/chemokines were measured in 42 individuals diagnosed with active pulmonary TB, 52% were HIV co-infected. Individuals, undergoing a 26-week standard TB treatment, were followed longitudinally over 18 months and measurements were associated with HIV status and rates of sputum culture conversion.

**Results:**

Plasma concentrations of interferon-inducible protein-10 (IP-10) and vascular endothelial growth factor (VEGF) were significantly reduced upon TB treatment, regardless of HIV status. By the end of treatment, IP-10 concentrations were significantly lower in HIV negative individuals when compared to HIV-positive individuals (p = 0.02). Moreover, in HIV negative patients, plasma VEGF concentrations, measured as early as 2-weeks post TB treatment initiation, positively correlated with the time of sputum conversion (p = 0.0017). No significant changes were observed in other studied immune mediators.

**Conclusions:**

These data suggest that VEGF plasma concentration, measured during early TB treatment, could represent a surrogate marker to monitor sputum culture conversion in HIV uninfected individuals.

## Introduction

*Mycobacterium tuberculosis* (Mtb) infection remains one of the world’s major causes of illness and mortality with an estimated 1.4 million deaths in 2010 [1]. Tuberculosis (TB) has become a global public health emergency, particularly in developing countries, where more than 90% of new TB cases and deaths occur [1]. Currently, monitoring of TB treatment relies mainly on sputum culture or smear microscopy status. Previous studies have shown an association between sputum culture conversion within the first 2 months of conventional TB therapy and non-relapsing cure">sputum culture or smear microscopy status. Previous studies have shown an association between sputum culture conversion within the first 2 months of conventional TB therapy and non-relapsing cure [2], [3]. However, sputum culture conversion at 2 months is not always predictive of cure in individual patients [4], [5]. Moreover, sputum culture status can be more difficult to monitor in patients with HIV co-infection [6] and is not applicable in the context of extra-pulmonary disease [7]. This is particularly a problem in areas of high prevalence of HIV and Mtb co-infection, such as South Africa, where approximately 60% of new TB cases are HIV seropositive. These limitations emphasize the need for additional surrogate biomarkers of response to TB treatment. In addition, the identification and validation of biomarkers that are predictive of risk of treatment failure or delayed response to TB therapy would be of value in case-management of patients and could contribute to improved outcomes. A number of recent studies have examined changes in host-specific markers over the course of TB therapy. For example, an association between sputum levels of IFN-γ and bacterial clearance has been demonstrated [8] and, while concentrations of plasma molecules such as soluble urokinase plasminogen activator receptor (suPAR) [9], soluble intercellular adhesion molecule type 1 (sICAM) [10], C-reactive protein (CRP) [11] and cancer antigen 125 (CA-125) [12] have been shown to correlate with disease severity and to decline with microbiologic response to treatment.

The goal of this study was to evaluate changes in plasma cytokines in patients undergoing treatment for TB as a means of identifying candidate host markers associated with microbiologic response to therapy. To this end, we measured 24 plasma mediators (endowed with pro-inflammatory, anti-inflammatory, chemoattractant or growth functions) before, during and after a 26-week course of standard TB treatment in 42 individuals, where over half were HIV-co-infected. Soluble mediator levels and their signature profiles were analyzed in relation to HIV-status and the time to sputum culture conversion as an indication of response to TB therapy.

## Results

### Studied Population

The studied population consisted of 42 individuals diagnosed with active pulmonary TB, 52% of whom were HIV co-infected (**Tables 1** **and** **2**). All patients presented with either positive sputum smear microscopy and/or positive culture for Mtb at enrollment. Drug susceptibility testing was performed at the start of treatment and resistance to isoniazid or rifampicin was a criterion for exclusion. There were no statistical differences in age and body mass index between HIV− and HIV+ individuals. Based on chest x-ray examination, HIV infected patients presented more frequently with minimal cavitary disease when compared to HIV uninfected patients (p = 0.048). This has been previously described [13] and could be related to compromised immune responses induced by HIV infection [14]. All patients completed the full course of DOTS (Direct Observed Treatment Short Course). Nine patients (3 HIV negative and 6 HIV positive subjects) remained sputum culture positive at 26 weeks and received an additional 26 weeks of treatment with the same drug regimen. By the end of treatment (26 or 52 weeks of DOTS), all patients had successfully converted to sputum culture negative. Studied individuals were divided into 3 groups according to the time of sputum culture conversion: ≤ 8 weeks, between 12 and 26 weeks and > 26 weeks after the initiation of TB therapy (**Tables 1** **and** **2**). Time of culture conversion was defined as the time of the first negative sputum culture with at least one subsequent negative culture and no subsequent positive results during the period of follow-up. No statistical difference was observed in the time of culture conversion between HIV− and HIV+ individuals.

**Table 1:** Clinical characteristics of HIV−TB+ individuals (n = 20).

			Clinical presentation of TB			Outcome	
HIV−TB+	Gender	Age at enrolment(years)	Culture at enrolment	Xray^a^	BMI	Culture at 26 weeks	Time of Conversion (wks)^b^
TB122	F	27	+	3	22.03	−	6
TB036	M	46	+	2	18.29	−	8
TB117	M	42	+	2	17.80	−	8
TB131	M	44	+	2	19.61	−	8
TB132	M	30	+	2	21.08	−	8
TB039	M	56	+	2	21.34	−	12
TB048	M	40	+	3	16.96	−	12
TB095	M	48	+	3	18.13	−	12
TB112	F	41	+	2	19.66	−	12
TB124	M	62	+	3	26.77	−	12
TB130	M	22	+	3	18.56	−	12
TB088	M	21	+	2	18.78	−	24
TB096	M	59	+	3	16.22	−	24
TB114	M	26	+	2	17.10	−	24
TB129	M	32	+	2	25.10	−	24
TB097	M	52	+	2	17.51	−	24
TB125	M	30	+	2	19.47	+	36
TB069	M	37	+	3	17.01	+	52
TB090	M	35	+	2	18.56	−	52
TB031	F	50	+	2	24.12	+	52
**Average**	**(M) 86%**	**40**	**(+) 100%**		**19.7**	**(+) 15%**	**21.1**
**Range**		[21–62]			[16.22–26.77]		[6]–[52]

M: Male, F: Female.

a : 1: No cavities ; 2: cavities <4 cm ; 3: cavities ≥4 cm.

BMI: Body mass index.

nd: not determined.

b : The time of conversion is defined as the first analyzed time point where culture was negative and remained negative at all following time points. Studied individuals were divided into 3 groups according to the time of sputum culture conversion: ≤ 8 weeks, between 12 and 26 weeks and > 26 weeks after the initiation of TB therapy.

**Table 2:** Clinical characteristics of HIV+TB+ individuals (n = 22).

HIV+TB+	Gender	Age at enrolment (years)	Culture at enrolment	Xray^a^	BMI	Culture at 26 weeks	Time of Conversion (wks)^b^
TBH041	M	32	+	2	18.37	−	4
TBH068	M	44	+	2	15.05	−	4
TBH084	M	39	+	2	19.03	−	4
TBH089	F	29	+	2	19.77	−	4
TBH037	F	51	+	1	20.26	−	6
TBH028	M	33	+	2	16.29	−	8
TBH080	M	24	+	nd	18.64	−	8
TBH113	F	52	+	2	19.81	−	8
TBH045	M	58	+	3	16.59	−	12
TBH079	F	23	+	2	19.29	−	12
TBH123	M	36	+	2	20.28	−	12
TBH033	F	39	+	2	nd	−	24
TBH034	M	33	+	2	nd	−	24
TBH035	M	44	+	2	nd	−	24
TBH093	F	21	+	1	21.93	−	24
TBH094	F	21	+	3	35.70	−	24
TBH074	M	36	+	2	17.63	+	36
TBH087	M	30	+	2	19.05	+	36
TBH081	M	31	+	2	28.01	+	52
TBH083	M	28	nd	2	nd	+	52
TBH135	M	41	+	3	nd	+	52
TBH091	F	38	+	1	17.91	+	52
**Average**	**(M) 64%**	**35.6**	**(+) 100%**		**20.21**	**(+) 27%**	**21.9**
**Range**		[21–58]			[15.05–35.70]		[4]–[52]

M: Male, F: Female.

a : 1: No cavities; 2: cavities <4 cm; 3: cavities ≥4 cm.

BMI: Body mass index.

nd: not determined.

b : The time of conversion is defined as the first analyzed time point where culture was negative and remained negative at all following time points. Studied individuals were divided into 3 groups according to the time of sputum culture conversion: ≤ 8 weeks, between 12 and 26 weeks and > 26 weeks after the initiation of TB therapy.

### Comparison of Baseline Plasma Cytokine Concentrations between TB+HIV− and TB+HIV+ Individuals

**Figure 1:**
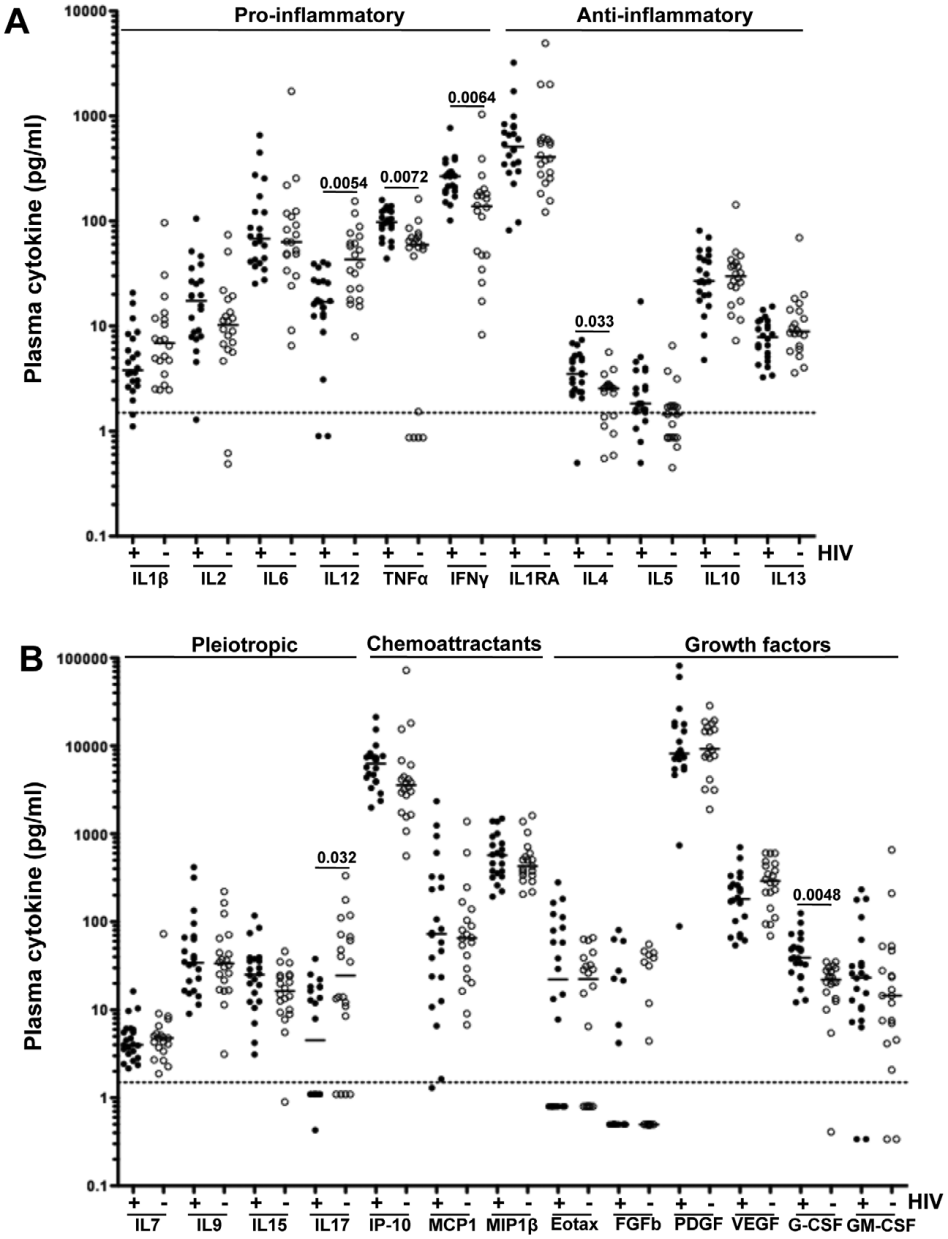
Comparison of baseline expression levels of soluble plasma mediators between TB+HIV − **and TB+HIV+ individuals.** (**A**) Mediators endowed with pro- and anti-inflammatory functions. (**B**) Mediators endowed with pleiotropic, chemoattractant and growth functions. Results are expressed as pg/ml of plasma. Open circles represent TB+HIV− subjects (n = 20) and black circles correspond to TB+HIV+ subjects (n = 22). Dotted lines represent the average limit of detection for all cytokines. Statistical comparisons have been performed using non-parametric Mann-Whitney U test and corrected for multiple comparisons using a false discovery rate (FDR) step down procedure.

**Figure 2:**
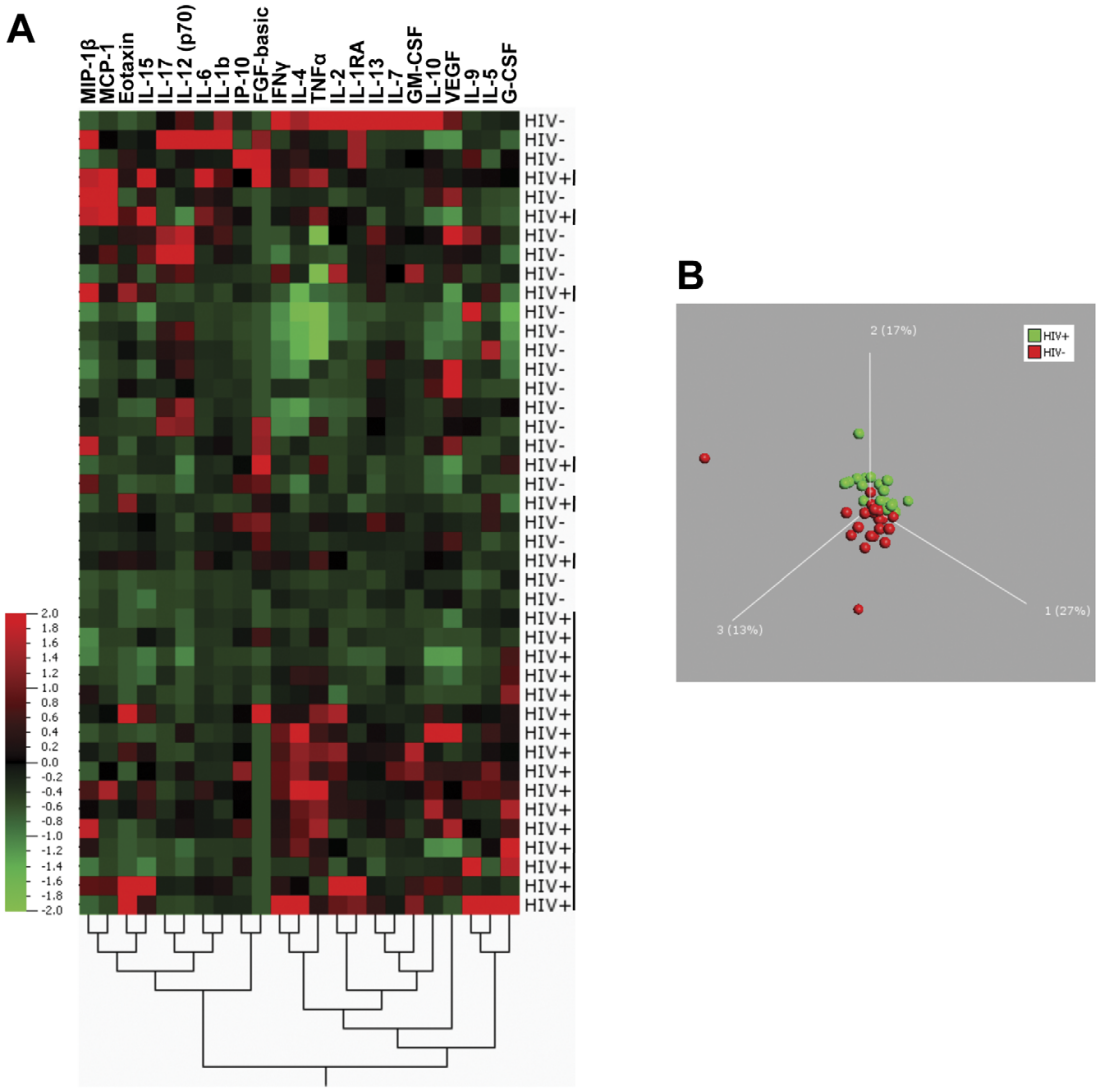
Expression profiles of baseline plasma cytokines in TB+HIV − **and TB+HIV+ individuals.** (**A**) Two-dimensional unsupervised hierarchical clustering of baseline cytokine profiles in TB+HIV− (n = 20) and TB+HIV+ (n = 22) subjects. The normalized values for each cytokine are depicted according to the color scale, where red and green represent expression above and below the median, respectively. (**B**) Three-dimensional representation of principal component analysis (PCA) of HIV− (red) and HIV+ (green) individuals. Each dot represents one subject based on values of all 23 cytokines studied. The percentage of variances is depicted on the 3 axes. The distance in space between each dot represents the relatedness between each individual.

Using the Luminex assay, we assessed the concentrations of 24 soluble plasma mediators at baseline and during treatment. Baseline was defined as the plasma sample collected before initiation of TB treatment. Comparison of baseline plasma concentrations of each of the 24 cytokines between TB+HIV− (n = 20) and TB+HIV+ (n = 22) individuals is shown in **Figure 1** **.** Significantly elevated concentrations of IL-4 (p = 0.033), G-CSF (p = 0.0048), IFN-γ (p = 0.0064) and TNF-α (p = 0.0072) and lower concentrations of IL-12(p70) (p = 0.0054) and IL-17 (p = 0.032) were observed in TB+HIV+ patients as compared to TB+HIV− patients. Of note, all reported p-values were adjusted for multiple comparisons, using a false discovery rate step-down procedure. The median absolute concentrations and interquartile range for each cytokine within the 2 groups are detailed in **Table S1**. To define if cytokine signature profiles were distinct between HIV+ and HIV− individuals, we performed unsupervised hierarchical clustering and principal component (PCA) analyses on log-transformed baseline cytokine concentrations. **Figure 2A** shows that 73% (16/22) of TB+HIV+ individuals grouped together, indicating that the majority of HIV infected individuals shared a specific cytokine expression pattern. The dendrogram (bottom of Figure 2A) shows the proximity between the different cytokines, suggesting that cytokines within each sub-cluster probably share the same origin, common transcriptional regulation and/or common function. These distinct cytokine profile signatures observed between TB+HIV− and TB+HIV+ individuals at baseline were further confirmed by PCA, based on the 24 studied cytokines (**Figure 2B**). PCA enables the discrimination and visual clustering of two or more classes, where individuals with similar patterns of cytokine expression are placed next to each other. As shown in **Figure 2B**, TB+HIV− and TB+HIV+ individuals visually segregate on a PCA plot, based on their baseline plasma cytokine expression patterns.

Overall, these results show that individuals with active TB disease who are co-infected with HIV show a distinct baseline (pre-treatment) cytokine expression pattern characterized by elevated plasma concentrations of TNF-α, G-CSF, IL-4 and IFN-γ and reduced plasma concentrations of IL-12(p70) and IL-17, when compared to individuals with active TB disease, but who are HIV uninfected.

### Longitudinal Assessment of Cytokine Expression Profiles Upon and after TB Treatment in TB+HIV− Individuals

**Figure 3:**
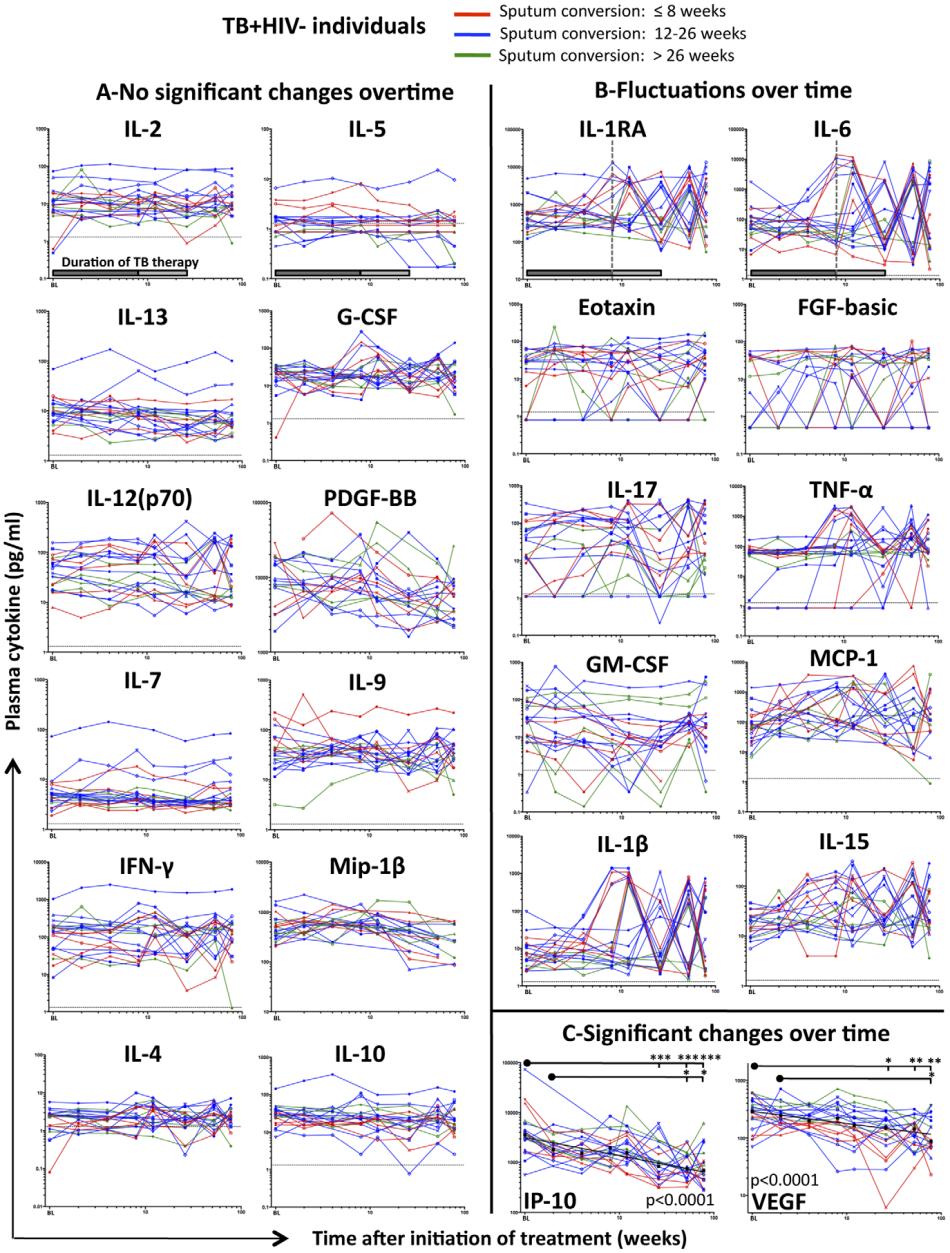
Longitudinal assessment of plasma cytokine expression levels before, during and after TB therapy in TB+HIV − **individuals.** The concentration of each cytokine has been measured at baseline, 2, 4, 8, 12, 26, 52, 78 weeks after the initiation of a 26- week treatment period. Individuals who presented with sputum conversion at ≤8 weeks are depicted in red, individual who presented with sputum culture conversion between 12 and 26 weeks are depicted in blue and individuals where sputum culture conversion occurred after 26 weeks of TB therapy are shown in green. (**A**) Plasma cytokine levels (IL-2, IL-5, IL-13, G-CSF, IL-12, PDGF-BB, IL-7, IL-9, IFN-γ, MIP-1β, IL-4 and IL-10) showing no significant change overtime. (**B**) Plasma cytokine levels (IL-1RA, IL-6, Eotaxin, FGF-basic, IL-17, TNF-α, GM-CSF, MCP-1, IL-1β and IL-15) fluctuating overtime. (**C**) Plasma cytokines (IP-10 and VEGF) presenting significant changes over time. Each dot represents one individual (n = 20). Results are expressed as pg/ml of plasma. Dotted lines represent the limit of detection for each cytokine. Gray shaded box (on the first graph) represents the duration of TB therapy; dark gray box depicts the intensive phase of treatment, including rifampicin, isoniazid, pyrazinamide and ethambutol, while light gray box corresponds to the second phase of treatment with rifampicin and isoniazid. Statistical analyses were performed using non-parametric one-way ANOVA Kruskal-Wallis Tests (*: p<0.05, **: p<0.01, ***: P<0.001). Numerical p-values, reflecting the overall changes in IP-10 and VEGF expression levels, have been determined using random-effects linear regression. The *x*-axis (time after the initiation of treatment in weeks) has been logged to allow better visualization of cytokine expression levels at early time points.

To define the impact of a 26-week course of TB therapy on cytokine expression profiles, we compared the concentrations of cytokines before (BL: Baseline), during (2, 4, 8, 12 and 26 weeks) and after (52 and 78 weeks) TB therapy. When analyzing the profile of each cytokine separately in TB+HIV− individuals (Figure 3), we identified three distinct response patterns: (i) No significant changes of the median cytokine concentrations over the studied period. Twelve cytokines followed this pattern including IL-4, IL-10, IL-2, IL-5, IL-7, IL-9, IL-13, G-CSF, IFN-γ, MIP-1α, IL-12(p70) and PDGF-BB (Figure 3A); (ii) Fluctuations in cytokine concentrations over the studied period (IL-1β, IL-6, IL-1RA, IL-15, IL-17, TNF-α, Eotaxin, FGF-basic, GM-CSF and MCP-1) (Figure 3B) and (iii) Significant decrease of the median cytokine levels over time. This latter profile was observed for IP-10 (chemoattractant) and VEGF (growth factor) (Figure 3C). Significant declines in IP-10 and VEGF concentrations during the course of TB treatment were confirmed using two different types of statistical analyses: A non-parametric one-way ANOVA Kruskal-Wallis test, assessing the change in cytokine concentrations between individual time-points, and random-effects linear regression evaluating the overall trend in log-transformed cytokine concentrations over time, with study participants matched at each time-point.

Using hierarchical clustering and PCA, we found that study participants did not have specific cytokine signature profiles upon TB treatment (data not shown), suggesting that large, often recurrent fluctuations that were observed for certain cytokines (such as IL-1β, IL-6, IL-15 or IL-17) did not appear to be related to TB treatment. Of note, when observed, fluctuations in cytokines concentrations were often detected after 8 weeks of treatment; a time coinciding with the end of the intensive phase of TB treatment, when pyrazinamide and ethambutol were discontinued. These variations did not associate with time to sputum culture conversion and were observed in individuals with early (<8weeks) or late (>26weeks) conversion (Figure 3B). Similar analyses were performed on the TB+HIV+ arm of the cohort and comparable results were obtained, where IP-10 and VEGF levels significantly decreased upon TB therapy (p<0.0001 and p = 0.04, respectively; **Figure S1**).

Taken together, these results show that TB treatment leads to a progressive decrease of IP-10 and VEGF expression levels in both TB+HIV− and TB+HIV+ individuals and no significant consistent changes in any others studied cytokines were observed upon TB therapy.

### Comparison of IP-10 and VEGF Down-regulation Rates Induced by TB Therapy in TB+HIV− and TB+HIV+ Individuals

**Figure 4:**
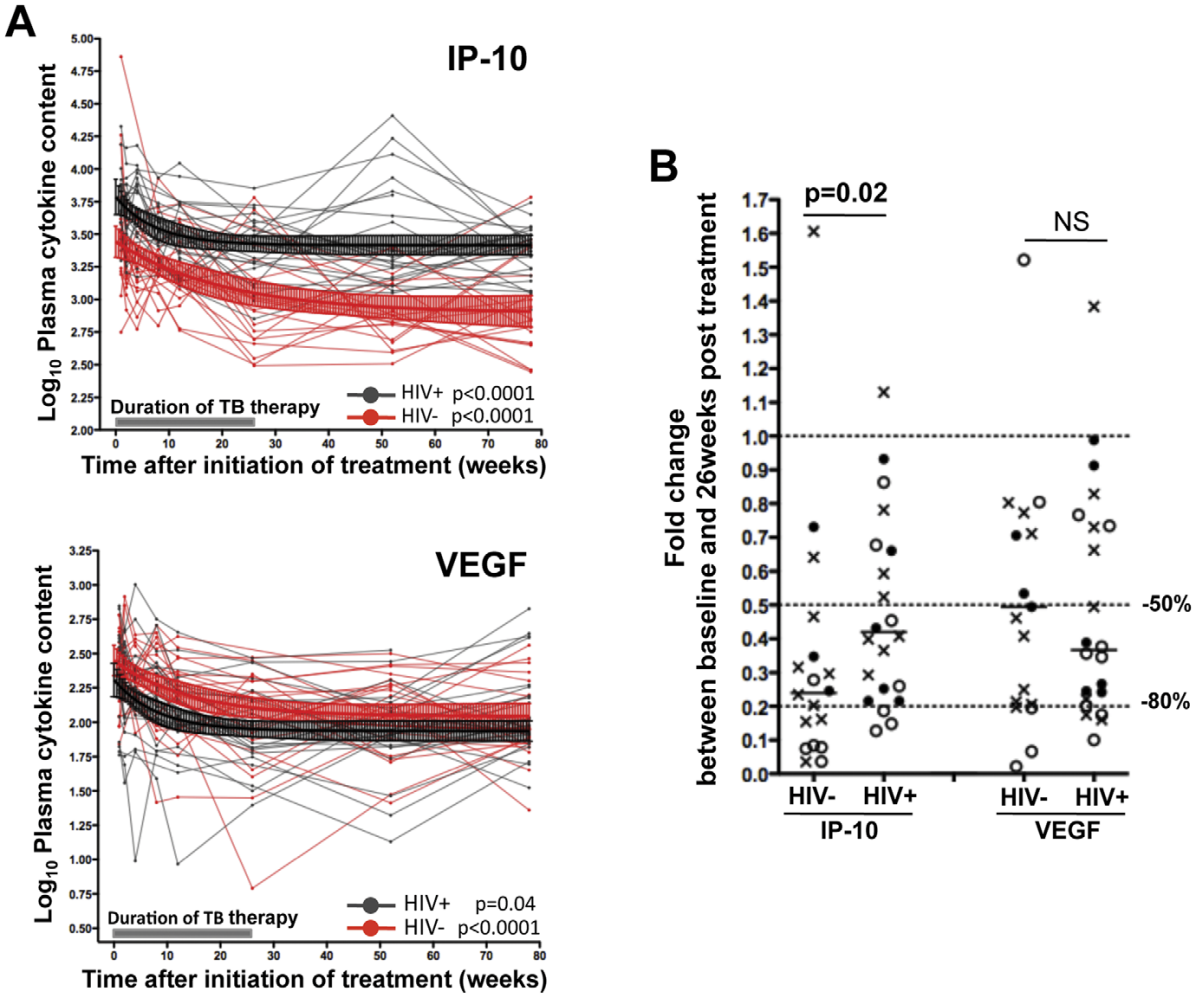
Comparison of the decrease rate of IP-10 and VEGF in TB+HIV − **and TB+HIV+ individuals upon TB therapy.** (**A**) Longitudinal plasma IP-10 and VEGF concentrations before, during and after TB therapy in TB+HIV− (Red) and TB+HIV+ (Black) individuals. Values were log-transformed. Median values with 95% CI for TB+HIV− (n = 20) and TB+ HIV+ (n = 22) individuals are depicted in red and black, respectively. A shaded grey box represents the duration of TB therapy. Statistical analyses were performed using random-effects linear regression. (**B**) Comparison of fold-changes in plasma IP-10 and VEGF concentrations between baseline and 26-week measurements (end of TB therapy) in TB+HIV− and TB+HIV+ subjects. Each dot represents one individual. Open circles correspond to individuals who presented with sputum conversion at ≤8 weeks, closed circles correspond to individuals who presented with sputum culture conversion between 12 and 26 weeks after TB therapy initiation and crosses identify individuals where sputum culture conversion occurred after 26 weeks post treatment. Doted lines depict 50% and 80% reduction of cytokine expression levels. Statistical analyses were performed using non-parametric Mann-Whitney U test.

To further characterize TB treatment-induced down-regulation of plasma concentrations of IP-10 and VEGF, we compared the kinetics of IP-10 and VEGF levels between TB+HIV− and TB+HIV+ individuals. In both groups, IP-10 and VEGF down-regulation followed a one-phase decay curve (**Figure 4A**). Upon TB therapy, the rate of decline in IP-10 level, between baseline and 26-week measurements, was significantly steeper in TB+HIV− patients when compared with TB+HIV+ patients (p = 0.02); where the median fold changes in IP-10 concentrations were 0.24 (IQR: 0.08–0.37) in TB+HIV− subjects and 0.41 (IQR: 0.23–0.67) in TB+HIV+ subjects (**Figure 4B**). Of note, fold changes in IP-10 concentrations between TB+HIV− and TB+HIV+ individuals were also significantly different between baseline and 2, 4, 52 and 78 weeks post TB therapy initiation [p = 0.03, p = 0.019, p = 0.012 and p = 0.045, respectively (data not shown)]. In contrast, the rate of decline in VEGF concentrations were comparable for both groups (**Figure 4A**), with no differences being apparent in the median fold changes between baseline and 26 week for either patient group (**Figure 4B**).

These data show that IP-10 down-regulation, in response to TB treatment, is more pronounced in TB+HIV− patients when compared to TB+HIV+ patients, suggesting that sustained viral replication possibly contributes to elevated concentrations of IP-10 in HIV infected TB patients.

### Relationship between Cytokine Concentrations and Time to Sputum Culture Conversion

**Figure 5:**
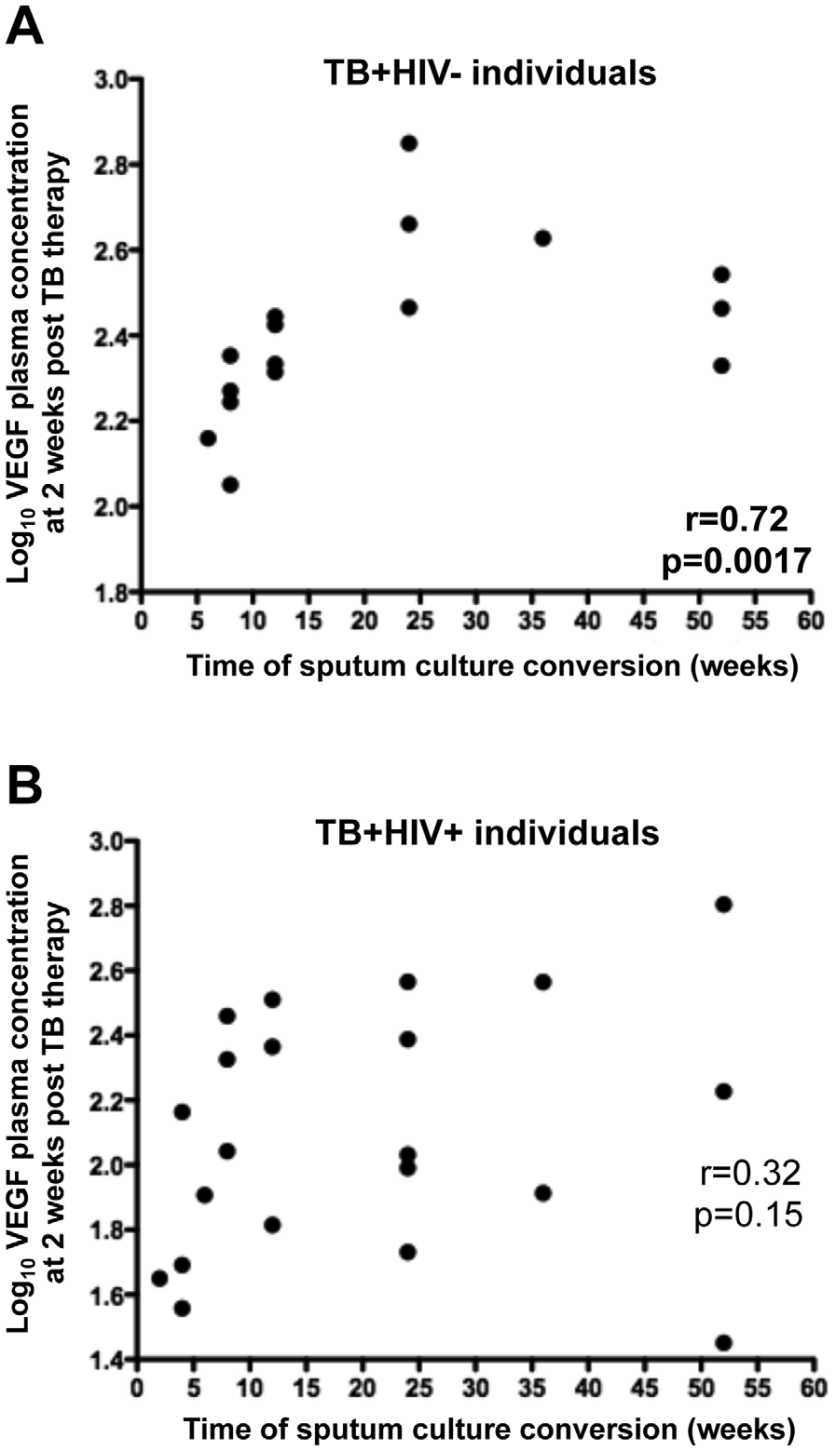
Correlation between plasma VEGF concentrations at 2 weeks post TB therapy initiation and time to sputum culture conversion. (**A**) TB+HIV− individuals; (**B**) TB+HIV+ individuals. VEGF concentration values were log-transformed. Statistical associations were performed by a two-tailed non-parametric Spearman rank correlation.

We next analyzed if any measured plasma factor could be potential candidates as surrogate markers for sputum culture conversion. Cytokine concentrations, at each time point, were correlated with the time to sputum culture conversion in TB+HIV− and TB+HIV+ individuals. Figure 5 shows that plasma VEGF concentrations, measured at 2 weeks after initiation of TB treatment, was positively associated with the time of sputum culture conversion in TB+HIV− individuals (r = 0.72, p = 0.0017, **Figure 5A**). Positive associations were also observed between VEGF concentrations measured at 4, 12, and 26 weeks post TB treatment and time of sputum culture conversion [p = 0.0032, p = 0.043 and p = 0.0036, respectively (data not shown)]. Of note, no association was found between baseline VEGF concentrations and the time of culture sputum conversion. These associations were not observed in the TB+HIV+ group at any time point (**Figure 5B**). Moreover, no associations were observed between any other cytokine measured and time of culture sputum conversion. These data suggest that plasma VEGF concentrations, measured as early as 2 weeks after the initiation of TB treatment, could predict bacterial clearance in HIV negative individuals and could be used as a prospective marker of time to sputum culture conversion.

## Discussion

In this report, we monitored the plasma cytokine environment during TB therapy and for 1 year afterwards in 42 individuals with active TB, 52% of whom were HIV positive. Since HIV infection is known to induce pro-inflammatory cytokine production, it is conceivable that HIV co-infected patients could possess distinct cytokine profiles, when compared with patients with TB only [15], [16]. Indeed we show, at baseline, that TB+HIV+ individuals displayed a different cytokine expression pattern when compared to TB+HIV− individuals, and co-infection was characterized by elevated IL-4, G-CSF, IFN-γ and TNF-α concentrations and decreased IL-12(p70) and IL-17 levels. This suggests that HIV co-infection could affect TB immune responses by causing dysregulation in cytokine production. Elevated TNF-α, in addition to playing a key role in granuloma formation [17], could also favour granulomatous necrosis increasing lung tissue damage [18], [19] and reduced IL-17 production could limit the recruitment of CD4 T cells into the lungs [20]. These data further emphasize the importance of considering concomitant infections when monitoring immune responses during TB, as they could considerably alter anti-bacterial immune responses.

Longitudinal assessment of the plasma cytokine profile showed that amongst the 24 mediators studied, only IP-10 and VEGF concentrations significantly decreased during the course of TB therapy in both TB+HIV− and TB+HIV+ individuals. We had expected that plasma concentrations of other cytokines (such as IFN-γ or TNF-α) would change in response to treatment-induced bacillary clearance, as bacillary load has been associated with changes in the immune response during TB treatment. For instance, it has been reported that IFN-γ levels in sputum closely correlated with mycobacterial clearance [8]. Moreover, Bekker *et al.* showed that initiation of TB treatment led to a transitory increase in TNF-α levels, while IFN-γ and IL-6 levels were progressively reduced, in individuals with severe TB [21]. The apparent stable cytokine expression levels observed for these molecules in the present study could reflect the compartmentalized production of cytokines, where cytokines are more likely to accumulate and decline at the site of infection in the lung.

Our longitudinal data revealed that some pro-inflammatory cytokines (such as IL-1β, IL-6, IL-15, and TNF-α) as well as IL-1Ra and MCP-1 displayed a fluctuating profile later during treatment. For most of the studied individuals, the occurrence of these fluctuations appeared to coincide with the transition from the intensive TB treatment phase to the continuation phase, when pyrazinamide and ethambutol were discontinued, or at the end of treatment. Although we do not have a substantiated explanation for this phenomenon, the timing of these observations suggests that alterations in drug regimens (2 months and 6 months of standard DOTS) may lead to changes in the regulation of some immune mediators. Evidence to support this interpretation was reported in a recent study demonstrating that pyrazinamide treatment can affect cytokine production in J774 cells and bone marrow-derived mouse macrophages and dendritic cells [22]. In addition, a number of studies have described the immunomodulatory effects of other antibiotics, in particular the quinolones and macrolides [23], [24].

Our data also show that, in response to TB therapy, only IP-10 and VEGF levels showed statistically significant changes in plasma in both TB+HIV− and TB+HIV+ infected individuals. IP-10 is a chemoattractant, facilitating the recruitment and trafficking of monocytes, T cells, NK cells and dendritic cells. IP-10 is produced by a variety of cells including neutrophils, monocytes, endothelial cells and fibroblasts in response to IFN-γ and TNF-α [25], [26], [27]. Thus IP-10 expression levels may reflect the extent of the ongoing pro-inflammatory immune response. Elevated expression of IP-10 has been described in both active TB [28], [29], [30], [31] and HIV infection [32], [33], [34]. It is commonly accepted that plasma IP-10 concentration is a reflection of TB disease activity [35], [36], [37]. Moreover, plasma IP-10 levels have been shown to decrease upon successful treatment [35], [38]. Hence, it has been speculated that IP-10 could be a better prediction marker of TB infection as compared to IFN-γ or TNF-α [28], [30], [36], [39]. The present data confirm that plasma IP-10 concentrations decrease significantly upon efficient TB therapy in both TB+HIV− and TB+HIV+ individuals and show that the rate of IP-10 down-regulation was lower in HIV co-infected subjects. It is plausible that ongoing HIV replication would lead to sustained immune inflammation and production of IFN-γ and TNF-α, fueling IP-10 production. Taken together, our results suggest that although IP-10 is not a specific marker for Mtb infection [26], monitoring IP-10 levels in active TB patients could be a valuable marker for clinical response and treatment outcome.

VEGF, endowed with potent angiogenic, lymphangiogenic and vascular permeability activities in endothelial cells, is up-regulated in response to tissue inflammation, hypoxia and pro-inflammatory cytokines (reviewed in [40], [41]). Thus, VEGF may influence leukocyte trafficking by playing a role in blood and lymphatic vessel function during inflammation [42]. Indeed, VEGF is chemoattractant for monocytes [40] and its over-expression in mouse models leads to abundant angiogenesis and inflammation [43]. Serum VEGF and angioprotein-2 levels have been shown to be associated with the severity of systemic inflammation in patients with inflammatory lung disease [44]. Although the specific role of VEGF in the pathogenesis of TB is unclear, it has been suggested that VEGF may participate in the neovascularization in granulomatous tissue [45], favoring the progression of chronic inflammation in association with pulmonary damage. In active TB, elevated plasma VEGF levels have been reported in several publications [46], [47], [48]; activated macrophages are the most likely source of VEGF in TB lesions [48]. In the present report, we observed that plasma VEGF concentrations decrease upon TB therapy with comparable rates in both TB+HIV− and TB+HIV+ individuals. Importantly, in HIV uninfected subjects, plasma VEGF concentrations, measured as early as 2 weeks after treatment initiation, positively associated with the time of culture sputum conversion. This suggests that as the bacillary load is controlled in the lungs, inflammation-induced VEGF production is reduced. Indeed, angiogenesis and chronic inflammation have been described as inter-dependent events [49]. The lack of association between VEGF and the time to sputum conversion in TB+HIV+ individuals may be due to chronic HIV-driven hyper-immune activation.

In conclusion, treatment-induced Mtb clearance, routinely assessed by sputum culture conversion is to date the principal measure of TB treatment success. However, more sensitive and rapid predictive assays are needed to improve clinical management of TB patients as emphasized in several publications [27], [50], [51], [52], [53]. Our data show that measurement of plasma IP-10 and VEGF concentrations could be a valuable adjunct to monitor TB treatment, and suggest that persistently elevated VEGF and/or IP-10 levels may reflect inefficient therapy or lack of treatment compliance. Further analyses in a greater number of individuals with appropriate control populations will be needed to validate the usefulness of this approach.

## Materials and Methods

### Studied Participants

Participants were recruited in two outpatient pulmonary TB treatment facilities in Durban (South Africa) between August 2000 and November 2001. Participants were eligible when ≥18 years of age and consenting to HIV pre- and post-test counseling and testing. Drug susceptibility testing was performed at the time of TB therapy initiation and individuals presenting with resistance to isoniazid or rifampicin were excluded from the study. Diagnosis of active pulmonary TB was based on symptoms, roentgenographic evaluation and epidemiologic history. All included patients presented with either positive sputum smear microscopy and/or positive culture for Mtb. Numerical score were used for grading Chest x-ray severity, where (1) corresponds to individuals presenting no cavities; (2) subjects with cavities < 4 cm and (3) individuals with cavities ≥ 4 cm. Plasma and sputum samples were collected at enrollment, 2, 4, 8, 12, 26, 52 and 78 weeks after the initiation of TB therapy. We studied a subset of 42 individuals who had samples collected at all time points. At the time of screening, 20 subjects were HIV-negative and 22 subjects tested positive for HIV. This study took place prior to the ARV roll-out program and patients did not receive any anti-retroviral therapy at that time. All HIV-infected individuals were viremic at the time of enrollment (median viral load: 18,860 copies/ml [IQR: 13,189–48,039]) with low CD4 counts (median: 282 cells/mm^3^ [IQR: 177–448]). Standard directly observed TB treatment (DOT) was started after enrolment and was in accordance with the South-African Guidelines for the management of TB [31]. All individuals were administered 4-drug fixed-dose combination tablets (rifampicin, isoniazid, pyrazinamide, ethambutol) for 8 weeks, followed by a 16 week-course of 2-drug fixed dose combination tablets (rifampicin, isoniazid). The ethics committees of Nelson Mandela Medical School and Witwatersrand University approved the initial study, with subsequent approval from the IRB of the University of Medicine and Dentistry of New Jersey, for the cytokine measurements on the plasma samples. All the subjects provided written informed consent for participation in this study.

### Bacteriological Assessment of Sputum Acid-fast-bacillus Smear and Sputum Culture

The diagnosis of TB was based on the detection of acid-fast bacilli under direct fluorescent microscopy and confirmed by culture on Lowenstein-Jensen media or MGIT culture system (Becton-Dickinson, Baltimore, USA) for baseline isolates of *Mycobacterium tuberculosis* and to determine TB outcomes at 8, 12, 26, 52 and 78 weeks. We defined culture conversion as the first negative sputum culture with at least one subsequent negative culture and no subsequent positive results.

### Multiplex Cytokine and Chemokine Analysis

Samples were kept at −80°C until use. Frozen plasma samples were quickly defrosted, spun down to remove clots. Samples were then passed through a Costar Spin-X Centrifuge Tube, 0.22 µm nylon filter, and diluted 1:4 with sample diluents provided in the Luminex kit. Cytokine levels were measured by multiplex bead array technology according to manufacturer’s instructions (Bulletin#10014905; Bio-Rad, Hercules, USA) and read on a luminometer (Luminex, Austin, USA). Bio-Plex Pro assays enabled us to quantify multiple protein biomarkers in a single well, using 12.5 µl of plasma. The use of magnetic (MagPlex®) beads allowed us to automate wash steps on a Bio-Plex Pro wash station offering greater reproducibility compared to vacuum filtration. Twenty-seven plasma mediators were evaluated. The panel included interleukin-1 beta (IL-1β), IL-1 receptor antagonist (IL-1RA), IL-2, IL-4, IL-5, IL-6, IL-7, IL-8, IL-9, IL-10, IL-12(p70), IL-13, IL-15, IL-17, IFN-γ (Interferon gamma), tumor necrosis factor alpha (TNF-α), macrophage inflammatory protein 1 alpha (MIP-1α), macrophage inflammatory protein 1 beta (MIP-1β), granulocyte colony stimulating factor (G-CSF), granulocyte macrophage colony stimulating factor (GM-CSF), eotaxin, fibroblast growth factor basic (FGF-basic), monocyte chemotactic protein (MCP-1), regulated upon activation normal T cell expressed and secreted (RANTES), IFN-γ-induced protein (IP-10), platelet derived growth factor BB (PDGF-BB) and vascular endothelial growth factor (VEGF). Of note, IL-8, RANTES and MIP-1α were excluded from the analysis, as measurements for these markers were outside the linear detection range of the assay.

### Unsupervised Hierarchical Clustering, Principal Component Analysis (PCA) and Statistical Analysis

Unsupervised hierarchical clustering of cytokine secretion of HIV positive and HIV negative individuals and generation of a heat map as well as PCA analysis were done using the Qlucore Omics Explorer software (Qlucore, Lund, Sweden). Statistical analyses were performed using GraphPad Prism version 5.0 (GraphPad Software, San Diego, USA) and STATA^TM^ version 10 (StataCorp, Texas, USA). Distribution of all variables was assessed by Shapiro-Wilk and Shapiro-Francia tests. Baseline comparisons of cytokine concentrations were performed using non-parametric Mann-Whitney U test and adjusted for multiple comparisons using false discovery rate step-down procedures [54]. For longitudinal measurements, soluble mediator concentrations were log-transformed and analyzed using one-way ANOVA Kruskal-Wallis test and Random-effects linear regression.

## Supporting Information

Figure S1 **Longitudinal assessment of plasma cytokine concentrations before, during and after the course of TB therapy in TB+HIV+ individuals.** The concentration of each cytokine has been measured at baseline, 2, 4, 8, 12, 26, 52, 78 weeks after the initiation of a 26- week treatment period. (A) cytokines showing no significant change overtime. (B) cytokines fluctuating overtime. (C) cytokines showing significant change overtime. Statistical analyses were performed using non-parametric one-way ANOVA Kruskal-Wallis Tests (*: p<0.05, **: p<0.01, ***: P<0.001). Numerical p-values, reflecting the overall changes in IP-10 and VEGF expression levels, have been determined using random-effects linear regression. The *x*-axis (time after the initiation of treatment in weeks) has been logged to allow better visualization of cytokine expression levels at early time points.
(TIF)

Table S1 **Baseline median plasma concentrations of cytokines in TB+HIV+ and TB+HIV− individuals.** Statistical comparisons have been performed using non-parametric Mann-Whitney U test and corrected for multiple comparisons using a false discovery rate (FDR) step down procedure. Interquartile Ranges (IQR) are shown in brackets.
(TIF)
