# Supplementary figures and images for: Effect of Standard Tuberculosis Treatment on Plasma Cytokine Levels in Patients with Active Pulmonary Tuberculosis
Source: PLoS One. 2012 May 14;7(5):e36886. doi: 10.1371/journal.pone.0036886 (PMC3351475; doi:10.1371/journal.pone.0036886)

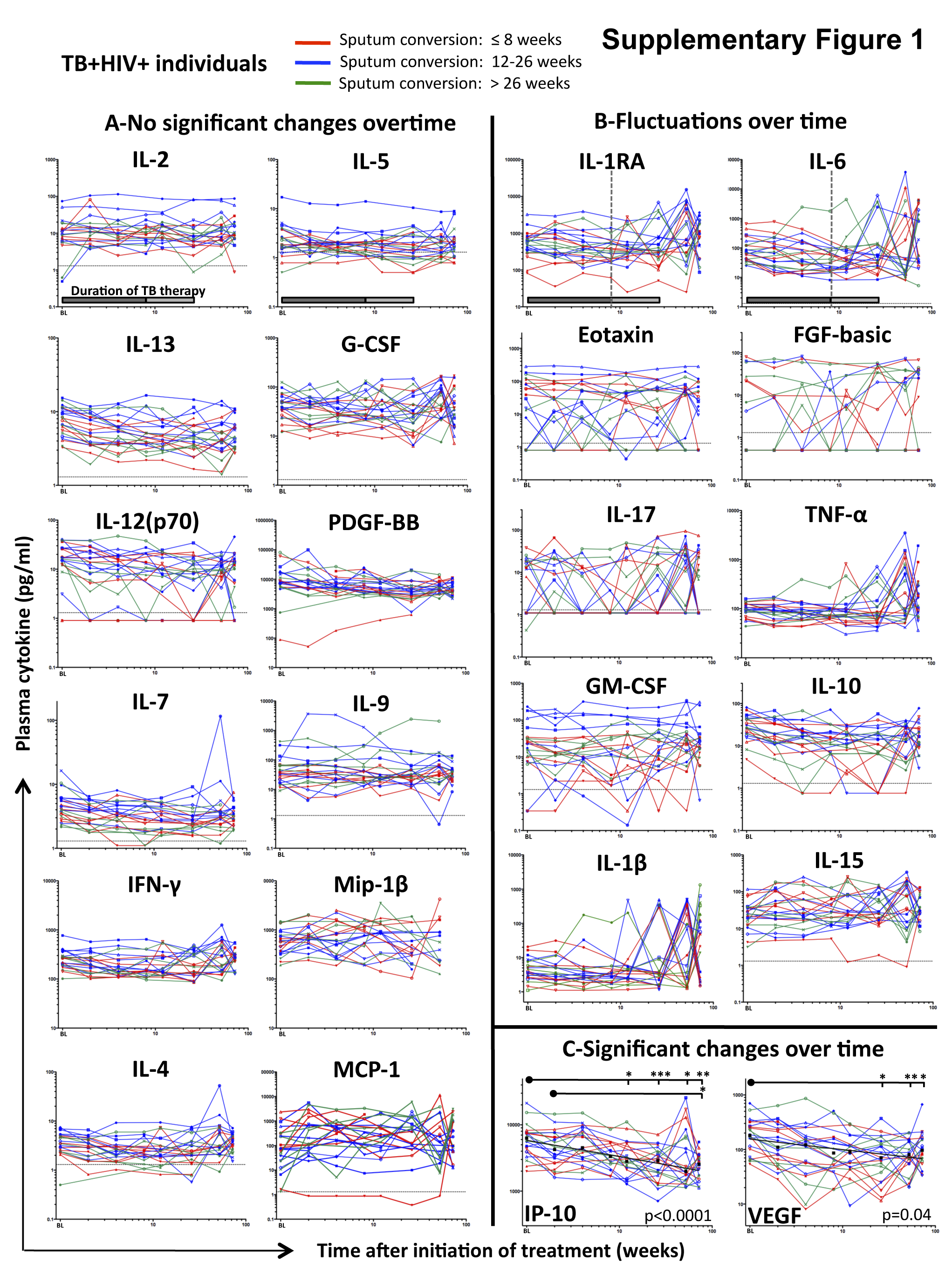

Supplement: Figure S1 — Longitudinal assessment of plasma cytokine concentrations before, during and after the course of TB therapy in TB+HIV+ individuals. The concentration of each cytokine has been measured at baseline, 2, 4, 8, 12, 26, 52, 78 weeks after the initiation of a 26- week treatment period. (A) cytokines showing no significant change overtime. (B) cytokines fluctuating overtime. (C) cytokines showing significant change overtime. Statistical analyses were performed using non-parametric one-way ANOVA Kruskal-Wallis Tests (*: p<0.05, **: p<0.01, ***: P<0.001). Numerical p-values, reflecting the overall changes in IP-10 and VEGF expression levels, have been determined using random-effects linear regression. The x-axis (time after the initiation of treatment in weeks) has been logged to allow better visualization of cytokine expression levels at early time points. (TIF) [file pone.0036886.s001.tif]

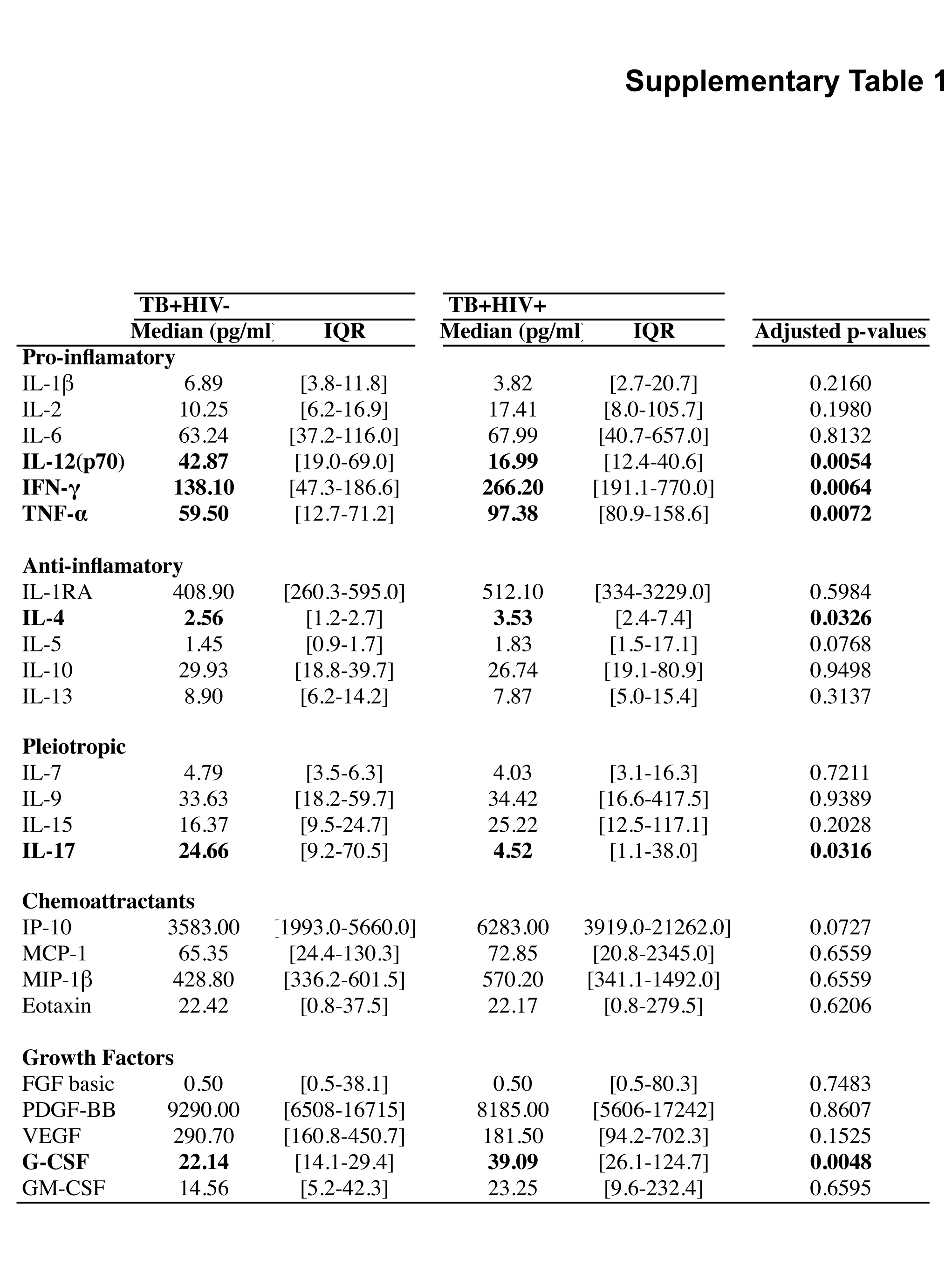

Supplement: Table S1 — Baseline median plasma concentrations of cytokines in TB+HIV+ and TB+HIV− individuals. Statistical comparisons have been performed using non-parametric Mann-Whitney U test and corrected for multiple comparisons using a false discovery rate (FDR) step down procedure. Interquartile Ranges (IQR) are shown in brackets. (TIF) [file pone.0036886.s002.tif]
